# The Managed Acquisition of Chemoresistance as an Informative Tool for Tumor Research

**DOI:** 10.3390/ijms262110400

**Published:** 2025-10-26

**Authors:** Tatyana A. Grigoreva, Daria N. Kindt, Aleksandra V. Sagaidak, Angelina A. Romanova, Vyacheslav G. Tribulovich

**Affiliations:** Laboratory of Molecular Pharmacology, St. Petersburg State Institute of Technology, St. Petersburg 190013, Russia

**Keywords:** chemoresistance, multidrug resistance, anticancer drug, cell culturing

## Abstract

The problem of acquiring chemoresistance by tumor cells is a growing concern for researchers as the effectiveness of diagnosis and treatment of primary tumors increases. To study the mechanisms of resistance, as well as to evaluate the effectiveness of new drugs, it is necessary to use adequate cell models. The review presents modern methods for obtaining chemoresistant cell lines used by researchers in such studies. It examines the most common cytostatics and targeted drugs, such as cisplatin, oxaliplatin, paclitaxel, doxorubicin, 5-fluorouracil, gemcitabine, gefitinib, bortezomib, erlotinib, and the monoclonal antibody cetuximab. Particular attention is paid to cell mechanisms activated due to drug resistance development and to methods of cell cultivation in the presence of drugs. The presented information provides an opportunity to discuss trends in the creation of chemoresistant cell lines for further research on resistance mechanisms and the development of new therapeutic strategies.

## 1. Introduction

Cancer is one of the main problems of modern medicine and is the cause of death of almost every sixth person in the world (16.8% of the total number of deaths) [1]. Such a high mortality rate in 90% of cases is due to the formation of tumor drug resistance [2]. In addition, the development of drug resistance to a single drug is often accompanied by resistance to drugs with different structures and mechanisms of action, called multidrug resistance [3].

One of the key causes of multidrug resistance in tumors is the malfunction of ATP-binding transport proteins, which belong to the ATP-binding cassette (ABC) family, also known as ABC transporters [4,5]. Proteins of this family are responsible for the absorption, distribution and excretion of various substrates from the cell—metabolic products, toxins, endogenous lipids, peptides, nucleotides, and sterols, as well as drugs [6,7]. However, a number of other mechanisms are known, such as improved DNA damage repair, suppression of apoptosis, alteration of the drug target, target gene amplification, and others, that often occur simultaneously [2,8].

As the effectiveness of diagnostics and therapy for primary tumors increases, the role of the chemoresistant phenotype that develops in response to various types of therapy becomes increasingly significant. Accordingly, the use of cell models that are able to recreate the features of tumors with drug resistance to various therapeutic agents is becoming an important element in the identification of effective drugs. Two-dimensional cell lines resistant to one or more drugs and 3D cultures based on them—organoids and spheroids—as well as microfluidic systems are used in such in vitro models [9,10,11,12,13]. Classic 2D cultures are the most widely used among them, which is explained by their availability, low cost of reagents and laboratory plastic, and ease of maintenance [14,15].

Regardless of the purpose of studying chemoresistant cells, they must first be isolated from a specific cell line resistant to a particular drug.

Establishment of drug–resistant cell lines is a complex, time-consuming process that requires careful selection of conditions. In this review, we systematize the data presented in the literature on methods for obtaining chemoresistant cell lines.

## 2. Application of Chemoresistant Cell Lines in Cancer Research

The development of chemoresistant cell lines still has specific scientific and applied significance, although it may also be an independent experimental task.

In general, cell lines obtained by cultivation in the presence of drugs can be used to study the mechanisms of chemoresistance and biomarkers that influence therapeutic efficacy or to assess the ability of any biologically active substances to overcome it [16]. In the first case, the focus may be on the drug specificity that provokes resistance (for example, to the effects of using paclitaxel in different cell lines) [5,17,18,19,20] or to cell-specific mechanisms (for example, MCF7 breast cancer cells) [21,22,23,24]. In the second case, cells serve as a tool for predicting the effectiveness of a therapy [20,25,26,27,28]. Accordingly, the tasks facing the researchers determine the specifics of the methods used to obtain chemoresistant cell lines.

### 2.1. The Study of Chemoresistance Mechanisms

For successful tumor therapy in clinical practice, it is necessary to take into account the cellular complexity of the disease and its dynamic and evolutionary features, which can both create obstacles and provide opportunities for successful treatment [29]. Anticancer therapy simultaneously creates conditions favorable for the selective growth of chemoresistant clones by destroying sensitive cells and disrupting their interaction. Currently, there are several theories describing tumor development, but the exact molecular mechanism underlying the development of drug resistance is still unknown [15].

Obtaining chemoresistant lines by using certain drugs makes it possible to identify specific mechanisms that are triggered in response to treatment. A comparative analysis of sensitive and resistant cell lines pairs makes it possible to study the molecular basis of resistance in detail. Among the most common mechanisms are:

- Overexpression of ABC transporters, such as P-glycoprotein (P-gp/ABCB1) and breast cancer resistance protein (BCRP/ABCG2), which reduce intracellular drug concentration to ineffective level due to efflux [4,5];
- Activation of anti-apoptotic signaling pathways, including proteins of the Bcl-2 family, which prevents the initiation of cell death in response to the drug [30];
- Induction of epithelial–mesenchymal transition, promoting metastasis and tumor progression, as well as the development of therapeutic resistance [31,32] and accompanied by the loss of epithelial markers, for example, E-cadherin;
- Structural changes in drug targets, for example, the predominance of tubulin isoforms that are less sensitive to taxanes [33].

Often acting synergistically, these mechanisms form a complex and multicomponent basis of chemoresistance, which emphasizes the need for an integrated approach to its study [34].

Furthermore, studies of differences in chemoresistant cells using omics methodologies make it possible to identify specific biomarkers that predict the effectiveness of therapy. In particular, various sets of miRNAs, including miRNAs of genes associated with apoptosis, DNA repair, or drug transport, correlate with resistance to 5-FU or oxaliplatin [35].

### 2.2. Search for Effective Anticancer Agents

Artificially developed chemoresistant cell lines play an important role in modern preclinical development strategies for anticancer agents. In vitro chemoresistance models enable researchers to identify compounds that overcome resistance in the nascent stages of drug development. This strategy is increasingly important with the development of personalized therapy and the rise of chemoresistant tumors.

Such models are used to test both new drug candidates and combination therapy regimens for previously known drugs. Such drugs can be based on components of natural origin or synthetic [36,37,38]. To overcome resistance to a particular drug, the use of sensitizing additives to the original drug is primarily considered, but a transition to fundamentally different treatment regimens is also possible [39].

Considerable attention of researchers is paid to the search for schemes to overcome P-gp-mediated multidrug resistance through the use of its inhibitors [40,41,42,43], as well as the search for alternative schemes based on targeted effects on pathways specific to a particular chemoresistant tumor. Thus, the use of the orally bioavailable targeted inhibitor ABT-263 reversed the resistance in the case of osimertinib-resistant subline HCC827/OR, where a significant increase in expression levels of Bcl-2 and Bcl-xL was noted [44].

### 2.3. 3D Cell Culturing

A separate area of chemoresistance research focuses on 3D structures. In such complexes, cancer cells resist therapy more effectively and exhibit their stem-like properties, as they reproduce the specificity of real tumors [45,46].

Although researchers often use two-dimensional (2D) cell cultures to study chemoresistance [9,47,48,49], they have a number of limitations related to the growth method and conditions that are atypical for a living organism. The inability to grow in three dimensions and the absence of a natural tumor microenvironment lead to changes in cell morphology [10,50]. Cell cultures cultivated on flat surfaces differ from in vivo tumors in their architecture, proliferation, and response to external stimuli [51,52,53]. In particular, they are more sensitive to the effects of drugs [54,55].

In order to make in vitro cell cultures more physiologically relevant, technologies for producing 3D cultures such as spheroids and organoids have been developed [15,56]. Spheroids are small spherical cellular formations spontaneously formed by self-assembly when creating specific non-adhesive growth conditions [9,57,58]. Spheroid cells more effectively form intercellular contacts, produce extracellular matrix, and exchange signals [59,60,61]. In turn, organoids are complex self-organizing cellular aggregates obtained from normal and cancer stem cells using special scaffolds and a particular nutrient medium [62,63,64,65].

Due to the more complex structural organization and reconstruction of the microenvironment, 3D models are closer to in vivo tumors than 2D models in terms of proliferation, tissue differentiation, and response to external stimuli [53,66,67]. Such systems are actively used for research purposes but have not yet found wide application in applied works in the search for anticancer agents. This is primarily due to the complexity and high cost of conducting such experiments, which are the main reason for excluding multiple drug candidate screenings.

## 3. The Main Drugs Associated with Research of Chemoresistant Cells

**Figure 1:**
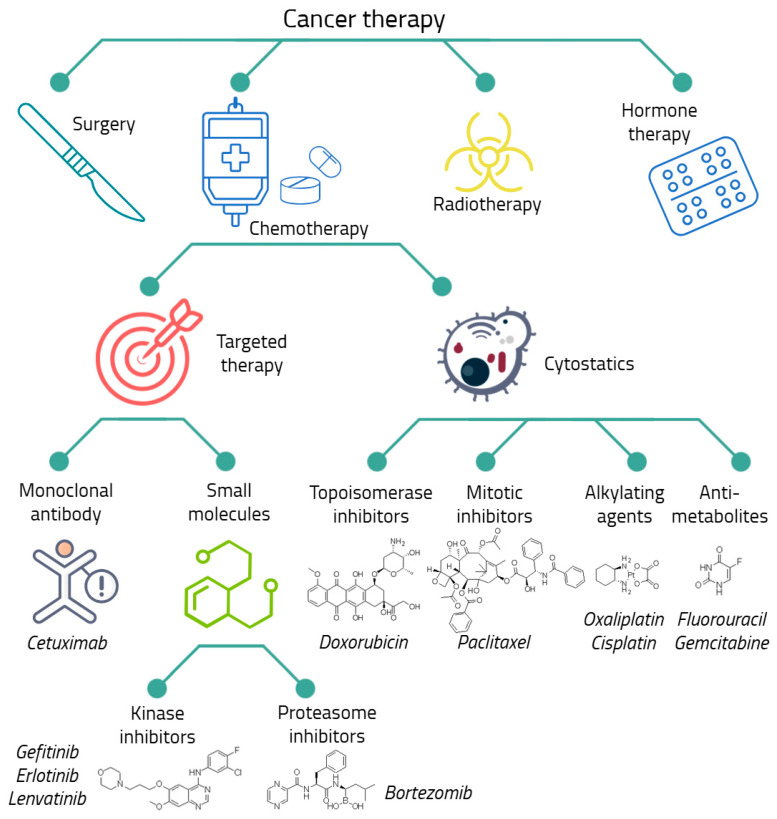
The main types of anticancer therapy. The considered drugs are presented.

Currently, there are a number of proven approaches to anti-cancer therapy, including surgical removal of the tumor, chemotherapy, radiotherapy, hormone therapy, immunotherapy, targeted therapy, etc. (Figure 1) [68,69]. To achieve the best clinical effect, different types of therapy are combined with each other; for example, surgical removal of a solid tumor is often combined with chemotherapy [70,71,72].

Despite the wide variety of treatments for malignant neoplasms, chemotherapy is the main and, in some cases, the only possible way of anti-cancer therapy [73,74,75,76]. Chemotherapeutic or cytostatic drugs disrupt the processes of growth, development, and division of cancer cells. Although this group includes agents that differ highly in their mechanism of action, the main classes of cytostatic drugs can be distinguished, such as alkylating agents, antimetabolites, topoisomerase inhibitors, and mitosis inhibitors [73,77,78,79,80].

The most dangerous side effects of cytostatic drugs are their toxicity to healthy cells and the development of tumor drug resistance [29]. To increase the effectiveness of chemotherapy and reduce side effects, targeted drugs are being actively developed and used. They act on specific molecular targets in cancer cells, stopping their growth and invasion [81]. Targeted drugs are small molecular compounds acting by various mechanisms. Their common feature is a direct interaction with a specific protein, leading to inhibition of signaling in cancer cells, which plays an important role in carcinogenesis [81,82]. They are highly selective and are successfully used in combinations with other methods, including chemotherapy [83,84]. An example of a targeted drug is the EGFR inhibitor gefitinib [85,86,87,88]. Nevertheless, targeted drugs are also involved in the development of tumor drug resistance [85,86,89].

Cytostatics occupy the main place among the drugs that attract the greatest interest from researchers in the context of the development of chemoresistance [90]. Cytostatics are widely used in chemotherapy and are believed to be a major contributor to the development of resistant tumors in patients. Such agents include cisplatin [91,92,93,94], oxaliplatin [39,95,96,97], paclitaxel (taxol) [17,89,98,99], doxorubicin (adriamycin) [100,101,102,103,104,105,106,107] and fluorouracil (5-FU) [39,108,109]. Nevertheless, in some cases, targeted drugs are also being investigated. Among them the greatest attention is paid to gemcitabine [39,110,111,112], bortezomib [113,114,115], erlotinib [86,116,117] and monoclonal antibody cetuximab [86,118]. These drugs are discussed in detail in Section 5.

## 4. Establishment of Cell Lines Resistant to Anticancer Drugs

Currently, more than 100 different cell lines resistant to cytotoxic and targeted drugs have been mentioned in literature. Resistant lines of various types of lung cancer and leukemia are most often obtained [90]. However, despite such a large amount of literature data and accumulated experience, there is still no single scheme for obtaining chemoresistant cell lines. In general, the basis for obtaining cell lines resistant to anticancer drugs is the routine treatment of cells with appropriate drugs for a certain period of time. Main parameters that must be determined prior to treatment include the drug concentration during initial cell treatment, the treatment cycle, and the treatment duration.

### 4.1. Determination of Anticancer Drug Concentration for Initial Cell Treatment

The concentration of anticancer drugs during initial cell treatment is an important parameter that directly affects the success of obtaining chemoresistant cell lines. It is obvious that using too high concentrations will lead to total cell death. At the same time, the concentration of the drug should be sufficient to activate the protective mechanisms of cell resistance. Accordingly, a necessary preliminary step is to establish the dependence of the survival and proliferation of a particular cell line on the concentration of a particular drug under conditions similar to selection. The most common is the use of colorimetric analysis methods, primarily MTT (for example, [119]), less often XTT [120] or SRB assay [121].

In most cases, half maximum inhibitory concentration (IC50) of the drug compound is used for the primary treatment of cells with anticancer drugs [86,106,110,122,123]. However, this is not a mandatory requirement; the initial concentrations of drugs can also be significantly higher or lower than IC_50_ [105,124,125]. IC_50_ values are also conveniently used to compare the sensitivities of parental and resistant lines, determining resistance index RI = (IC_50_ for resistant cell line/IC_50_ for parental cell line) [126,127]. Depending on the duration and cultivation scheme in the presence of drug compounds, the resulting cell lines may be resistant to final concentrations of the drugs used, exceeding the IC_50_ value of the parental lines from several to one hundred and a half times [5,99,122,124,128]. RI in the range 2–10 indicates moderate resistance, while RI above 10 indicates strong drug resistance [126]. However, it should be borne in mind that comparison of RI values is only valid for the same drug or for the same line and using identical viability assessment conditions.

### 4.2. Total Duration of Cell Culture with Anticancer Drugs

The duration of cells in the presence of anticancer drugs is an important parameter of the process of obtaining chemoresistant cell lines. Depending on the tasks, it may take months for a researcher to obtain a line with the desired properties. The degree of cellular adaptation to the drug and, accordingly, the degree of chemoresistance developed directly depends on this parameter [129]. Cell culture in the presence of an anticancer drug can be both short-term and long-term.

Short-term treatment may be appropriate in the context of reproducing a specific therapeutic regimen or as an illustration of the drug’s potential to develop resistance. In this case, short-term cell culture in the presence of an anticancer drug is carried out until their proliferative ability is restored. On average, this process can take 2–3 weeks from the first treatment [122,124]. However, it should be borne in mind that short-term treatment can lead to incomplete adaptation of cells to the drug and weak activation of cellular defense mechanisms, which does not allow obtaining an adequate cellular model. With such “insufficient” treatment, a subsequent increase in the concentration of the anticancer drug can lead to cell death instead of adaptation [129].

Long-term cell culturing in the presence of an anticancer drug is carried out until they achieve stable proliferation. Depending on the cell type, the mechanism of drug action, and its concentration, this process can take from one month to one and a half years, during which the concentration of the drug is gradually increased as the cells adapt [89,130]. This approach allows achieving maximum activation of the cellular defense mechanisms against the anticancer drug [129].

### 4.3. The Schedules of Anticancer Drugs

**Figure 2:**
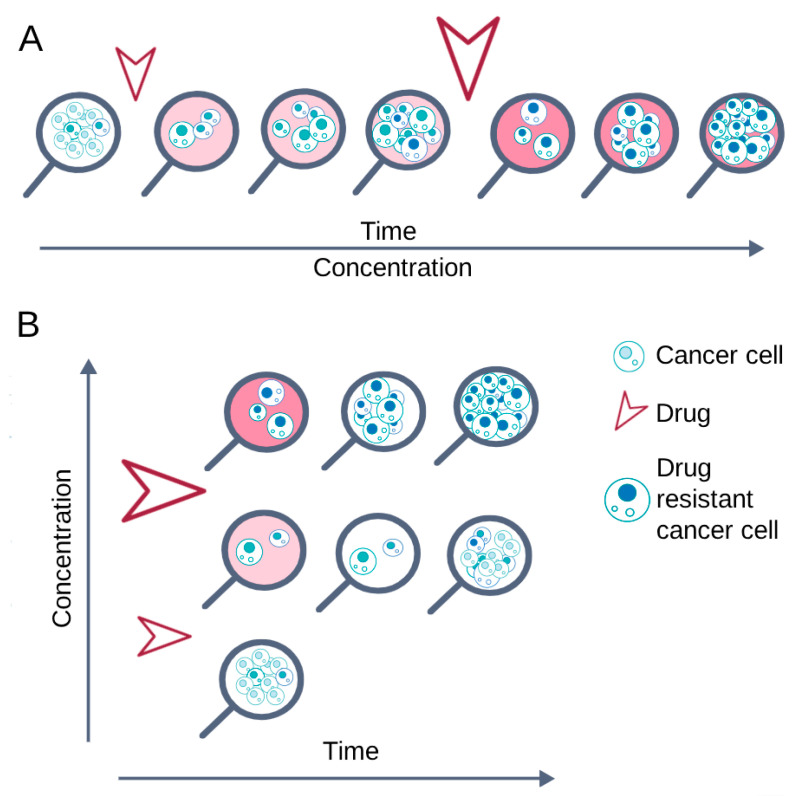
Basic cell treatment schemes to obtain chemoresistant cell lines. The arrows indicate the drug addiction. (**A**) Continuous treatment: the drug is constantly present in the medium; its concentration gradually increases as the cells adapt. (**B**) Episodic treatment: the drug is added for a certain time, then the cells are restored in a drug-free medium, and finally the drug is added again.

The scheme of cell culturing in the presence of an anticancer drug directly affects the formation of cellular mechanisms of chemoresistance. Two opposing schemes can be distinguished: episodic and continuous treatment (Figure 2). The differences between these schemes are due to the cell culture conditions that are maintained until cell growth and proliferation rates are restored [130].

With continuous treatment, cells are constantly exposed to the anticancer drug until they restore vitality and normal proliferation; after that, the concentration of the substance is increased [94,101,104,109,110]. This cultivation scheme promotes the survival of cells that can quickly activate defense mechanisms and, conversely, the death of cells that cannot adapt to adverse conditions.

During episodic treatment, cells are exposed to an anticancer drug for a short time, from several hours to several days, after which the drug is completely removed from the culture medium. Re-treatment with the drug is carried out only after the cells restore their viability and normal proliferation [17,87,91,108]. Removing the drug from the medium allows cells of various subpopulations, including the most sensitive ones, to develop resistance mechanisms before the next treatment. The cycle of treatment with the drug and its removal from the culture medium is repeated until the cells restore viability and normal proliferation in the presence of the drug used [93,98,100,108].

Episodic treatment (short-term drug pulses) mimics clinical bolus therapy, while continuous treatment essentially corresponds to continuous infusion of the drug [95,115].

## 5. Trends in the Development of Chemoresistant Cell Lines

In this review, we examined published data describing the establishment of cell lines resistant to the most commonly used anticancer drugs [90]. Methods for obtaining resistant cell lines depending on the specific drug are shown in Table 1.

While the choice of drug is generally determined by its applicability of a treatment to specific tumor types (for example, gefitinib against lung cancer or platinum-based drugs for ovarian cancer), the rationale for selecting a specific scheme is not always explicitly stated in the literature. Nevertheless, general trends can be noted. Thus, continuous treatment appears to be more commonly used in case of chemotherapy drugs (gemcitabine) and targeted drugs (cetuximab and lenvatinib). For 5-FU, doxorubicin, and gefitinib, episodic treatment is rather a rare exception. However, at the same time, an episodic type of drug treatment is mainly used to obtain cisplatin-resistant cell lines [91,92,93,123,131,132,133]. Thus, the researcher can choose a scheme for obtaining a chemoresistant cell line based on both the general trends shown in the table and their own preferences, depending on the tasks to be solved and the time frame.

The oxaliplatin example is illustrative, in which an episodic treatment was used in comparison with a continuous one [95]. The approach presented in this article allows a realistic assessment of the differences in treatment effectiveness. The authors showed that 7 months of continuous treatment can achieve significantly better results compared to 4-h pulses every passage. Notably, this resulted in a two-fold difference in RI for oxaliplatin in ovarian carcinoma cells.

In general, continuous treatment seems preferable, as it allows for the effective selection of cells with greater drug resistance compared to episodic treatment. At the same time there are no significant differences in the activated mechanisms between these treatment schemes. In addition, continuous treatment is more universal, since it does not involve variability in the frequency or duration of individual pulses.

Studies of different drugs on a single cell line identified mechanisms inherent to the line [39]. In particular, in a gastric adenocarcinoma cell line, any drug provoked the activation of the efflux pump (MRP↑), although 5-FU also activated P-gp, and oxaliplatin and paclitaxel suppressed the tumor suppressor DAPK2. Similarly, MCF7 adapts to taxol and doxorubicin by overexpressing specific transporters [98,99,105].

### 5.1. Cytostatics

It is evident that a mechanism of drug action should largely determine the cellular response, including the mechanisms of successful tumor cell defense. Thus, ovarian and colorectal cells reacted to oxaliplatin in a similar way, demonstrating a decreased uptake by one of the OCTs or CTR1, while the ATPases contributed to the efflux of platinum derivatives [95].

The cytotoxic drugs cisplatin and oxaliplatin belong to the class of alkylating agents. These drugs transfer alkyl groups to guanine residues of DNA, forming DNA adducts, DNA cross-links, and DNA strand breaks [77]. In addition, platinum-based drugs, including cisplatin and oxaliplatin, cause the formation of reactive oxygen species and cell death as a result of oxidative stress [145]. Accordingly, in the case of cell survival, they are expected to show changes in both the amount of DNA and its sequence, which contributes to the cell cycle arrest at different phases [39,91,93,94,123,132], and DNA repair activated by ERCC1 [92,95].

Paclitaxel belongs to the class of mitosis inhibitors. It binds to the tubulin, prevents the movement and functioning of microtubules, disrupts the formation of the mitotic spindle, and, as a result, suppresses the mitosis of tumor cells [78]. Overcoming such an effect in resistant cells, regardless of the drug treatment scheme, is facilitated by cell cycle arrest in the G0/G1 or G2/M phases [39,98], as well as the activation of drug efflux via P-glycoprotein or related MRP/LRP proteins, which in the case of paclitaxel was noted for all cell types [17,39,89,98,99,135].

Doxorubicin is an inhibitor of topoisomerase, an enzyme involved in DNA replication. Binding to topoisomerase eventually leads to DNA double-strand breaks [79]. Doxorubicin also causes the generation of reactive oxygen species and cell death from oxidative stress [146]. This drug has several cytotoxic mechanisms of action, which also allow it to be classified as an alkylating agent and an inhibitor of mitosis [73]. Nevertheless, all its potential effects are effectively overcome by P-gp-mediated efflux [100,102,103,105,106,107,136,137].

5-Fluorouracil (5FU) and gemcitabine are antimetabolites. Due to structural similarity to purines and pyrimidines, drugs of this class integrate into DNA, suppress its synthesis, and cause DNA strand breaks [80].

Accordingly, cells manage to adapt to 5-FU independently of the drug treatment scheme by activating the Twist transcription factor [108,139], which is associated with tumor stage, grade, and poor prognosis in multiple cancers [147]. Gemcitabine also inhibits the DNA polymerase and ribonucleotide reductase enzymes [148]. Thus, the activation of ribonucleotide reductase RRM1/2 [112,140] protects cells by providing DNA repair. Cell cycle arrest, as well as efflux activation, is also effective in cases of antimetabolites resistance [39,109,110].

In general, ABC family transporters, particularly P-gp, are a universal mechanism that is activated in cells in response to cytostatics, which has been noted in the vast majority of studies.

### 5.2. Targeted Drugs

It is known that the main mechanism of cellular defense against cytotoxic drugs is to prevent the accumulation of dangerous drug concentrations. In the case of targeted drugs, a change in the affected mechanism has a greater effect, which has been shown not only for modulators of the epidermal growth factor receptor (EGFR) but also for other agents [89].

EGFR is a central regulator of proliferation and progression in human cancers, as its ligand EGF regulates epithelial cells, which are known as the carcinoma precursor cells. EGF binding activates the EGFR tyrosine kinase, leading to autophosphorylation of tyrosine residues and activation of several signaling cascades, including MAPK, AKT, and STAT [149]. Cetuximab is a monoclonal antibody that blocks EGFR from outside the cell, while erlotinib and gefitinib are small molecular tyrosine kinase inhibitors that block EGFR signals inside the cell. It is quite predictable that the target drug loses its effectiveness due to any changes both in the structure of the target protein or in the mechanisms associated with it [85,86,144]. In the case of EGFR, signaling changes that promote tumor cell survival are associated with epithelial-to-mesenchymal transition, which is mediated by decreased E-cadherin levels [150]. Cell cycle arrest protects cells against apoptosis [87].

Both tumor and non-tumor cells become insensitive to the proteasome inhibitor bortezomib in the presence of mutations in the β5 subunit of the proteasome, to which the inhibitor is intended to bind [151,152,153]. Upon successful binding, bortezomib-mediated inhibition of proteasomes leads to the accumulation of a wide variety of proteins in cells. In turn, changes in mitochondrial metabolism (Bcl/Mcl) help cells adapt to proteotoxic stress [115,154], while increased expression of p-ERK and p-p65, which are involved in the NF-κB pathway [114,155], may exert an anti-apoptotic effect.

## 6. Conclusions and Perspectives

The problems of effective treatment of chemoresistant tumors are associated with a high degree of complexity and diversity of mechanisms for the development of cell resistance to antitumor drugs. Overcoming these problems requires a deep understanding of the processes underlying the development of resistance, as well as the mechanisms of their regulation at the molecular and cellular levels. Accordingly, an important aspect is the use of adequate cellular models that allow modeling the dynamics of the development of resistant phenotypes and studying their biological properties. Such models provide an opportunity to carry out an early-stage assessment of the potential of new drugs and also allow identifying the mechanisms that contribute to the emergence and progression of chemoresistance. In the context of modern pharmacology and oncology, the use of cellular systems that are as close as possible to clinical situations will facilitate a more accurate assessment of the effectiveness and risks of developing resistance to new drugs.

To date there are no generally accepted conditions for obtaining reproducible universal models in different laboratories, which would provide reproducible and comparable results. Treatment of the cells with anticancer drugs can be episodic or continuous, short-term or prolonged. The concentration of the drug and the cell culture conditions may also vary. Accordingly, a step towards obtaining more reproducible and relevant results would be the adoption of generalized protocols that could be used by researchers from different organizations and countries. As noted above, under current conditions, it is not possible to use even the resistance index (RI) for comparison, since all data are obtained under different conditions and, with rare exceptions (for example, [95,115,156]), the authors do not provide justification for the selected parameters.

To make the cell models clinically relevant, the use of 3D culture techniques can be considered. In the only study that investigated the development of resistance directly in gastric cancer organoids, 5-FU was used for 72 h, and the authors managed to achieve an almost 10-fold increase in IC_50_ [157]. 3D cultures are not suitable for prolonged studies due to their low stability [15]. However, these models are successfully used to study the mechanisms of resistance by reproducing the three-dimensional structure of in vivo tumors, which are characterized by diffusion gradients of substances and oxygen. [158]. Hypoxia and the uneven distribution of drugs within in vivo tumors contribute to the development of specific drug resistance mechanisms [158] that can only be replicated under appropriate conditions. Such models can be derived from 2D cultures with induced resistance, from the tissues of a patient’s drug-resistant tumor, or by treating three-dimensional systems with a drug.

Cell lines with induced drug resistance to various antitumor drugs remain a convenient, widely used model for studying the mechanisms of tumor cell resistance to drug compounds and preclinical evaluation of the effectiveness of new anticancer agents. Cell lines resistant to cytostatic drugs such as cisplatin or doxorubicin are most often obtained. Targeted drugs are less commonly considered. Nevertheless, given the increasing role of chemoresistance in the problems of successful cancer therapy, it seems advisable to expand the pool of similar trials to other drugs used in anticancer therapy, as well as ongoing preclinical and clinical trials.

## Figures and Tables

**Table 1:** Methods for obtaining resistant cell lines depending on the specific antitumor drug.

Drug		Drug Treatment Scheme	Tumor Type	Parental Cell Line	Activated Mechanisms of Drug Resistance *	Reference
Platinum-based alkylating agent	Cisplatin	Continuous	Gastric adenocarcinoma	OCUM2M	Modal chromosome number ↓, DNA index ↑	[94]
		Episodic	Colorectal carcinoma	HCT116	Migratory ability ↑	[131]
			Hepatocellular carcinoma	SK-Hep1	P-gp and MRP1 protein level ↑, G2/M cell cycle arrest	[93]
			Neuroblastoma	TGW	Alterations in the DNA sequence throughout the entire genome	[132]
			Osteosarcoma	SOSP9607	MRP1 and MRP2 mRNA ↑, G0/G1 cell cycle arrest	[91]
			Ovarian cancer	NOY1	p-Akt, p-Bcl-2 and GSTA1 ↑	[133]
			Squamous cell carcinoma	EC109	G0/G1 cell cycle arrest	[123]
	Oxaliplatin	Continuous	Colorectal cancer	SW620	E-cadherin ↓, Vimentin ↑	[96]
				HCT8 and HT29	CDK1 protein and mRNA ↑	[97]
				THC8307	*DDB1* and *RPA1* ↑, *STK17A* and *BNIP3* ↓, RAP1B and RGS4 ↓	[134]
			Gastric adenocarcinoma	OCUM2M	DAPK2 ↑, G2/M cell cycle arrest	[39]
			Ovarian carcinoma	A2780	hCTR1, OCT1, ERCC1, ATP7B and ALDH1L2 ↓; ALDH1A2 ↑	[95]
			Colorectal cancer	LoVo-92	hCTR1, OCT1, OCT2, OCT3, ERCC1 and ALDH1L2 ↓; ATP7A, ATP7B and ALDH1A2 ↑	[95]
				LoVo-Li	hCTR1, OCT2, OCT3, ATP7A, ATP7B and ALDH1L2 ↓	[95]
		Episodic	Ovarian carcinoma	A2780	hCTR1, OCT1, ATP7A, ATP7B and ALDH1L2 ↓; ERCC1 and ALDH1A2 ↑	[95]
			Colorectal cancer	LoVo-92	hCTR1, OCT1, OCT2, OCT3, ERCC1 and ALDH1L2 ↓; ATP7A, ATP7B and ALDH1A2 ↑	[95]
			Colorectal cancer	LoVo-Li	hCTR1, OCT1, OCT2, OCT3, ERCC1, ATP7A, ATP7B and ALDH1L2 ↓; ALDH1A2 ↑	[95]
Natural mitosis inhibitor	Paclitaxel (taxol)	Continuous	Breast cancer	SK-BR3	P-gp, BCRP, ABCC3, ABCC4 protein and mRNA ↑	[99]
				MCF7	P-gp, BCRP, ABCC3, ABCC4 protein and mRNA ↑	[99]
				MCF7	Hsp90, pre-dermcidin and actinin ↓	[135]
			Colorectal cancer	HCT116	P-gp ↑	[89]
			Gastric adenocarcinoma	OCUM2M	MRP ↑, DAPK2 ↓, G2/M cell cycle arrest	[39]
		Episodic	Breast cancer	MCF7	P-gp, LRP and GST-π ↑; G0/G1 cell cycle arrest	[98]
			Prostate cancer	DU145 and PC3	P-gp ↑	[17]
				VCaP, PC3 and DU145	LARP1 and CCND1 ↑	[124]
Topoisomerase inhibitor	Doxorubicin	Continuous	Breast cancer	MCF7	P-gp and BCRP ↑, procaspase-9 ↓	[105]
			Cholangiocarcinoma	QBC939	P-gp ↑, G2/M cell cycle arrest	[136]
			Colorectal cancer	HCT15	P-gp and MRP mRNA ↑	[102]
				LoVo	P-gp and COX-2 ↑	[137]
			Kidney cancer	RCC8701	P-gp, GST-p, and topoisomerase II mRNA ↑; GSH and G-6-PDH ↑	[101]
			Leukemia	BFTC905	227 genes ↑, 213 genes ↓	[104]
				KK47	P-gp ↑	[106]
			Lung cancer	SBC3	P-gp and GST ↑	[103]
			Osteosarcoma	MNNG/HOS	P-gp ↑, MRP ↓	[107]
				MG63	P-gp ↑	[107]
			Prostate cancer	DU145	ABCG4 ↑	[138]
		Episodic	Osteosarcoma	SAOS2	P-gp and MRP mRNA ↑	[100]
Antimetabolite	5-Fluorouracil (5-FU)	Continuous	Breast cancer	MDA-MB231	P-gp and BCRP ↑	[109]
			Gastric adenocarcinoma	OCUM2M	MDR1, MRP and DPD ↑, G2/M cell cycle arrest	[39]
			Hepatocellular carcinoma	HLF	*CDH1* and *TWIST1* ↑, *MRP5* ↓	[139]
		Episodic	Squamous cell carcinoma	HSC2 and HSC4	N-cadherin and Twist ↑, E-cadherin ↓	[108]
	Gemcitabine	Continuous	Gastric adenocarcinoma	OCUM2M	MRP ↑, G2/M cell cycle arrest	[39]
			Cholangiocarcinoma	KKU-M139 and KKU-M214	MRP1, Bcl-2, MMP-9 and uPA ↑; G2/M cell cycle arrest	[110]
			Lung cancer	CL1-0	p-PI3K/PI3K, p-AKT/AKT, and p-NF-κB/NF-κB ↑	[111]
			Pancreatic cancer	PANC1	NT5, RRM1 and RRM2 ↑	[112]
				PANC1 and Capan1	SLC38A5 and RRM1 ↑	[140]
				BxPC3	RUNX1 ↑	[141]
	Combination of docetaxel, cisplatin and 5-FU	Continuous	Head and neck cancer	Hep2	Survivin, CTR1, TS and ERCC1 ↑; G2/M cell cycle arrest	[92]
				CAL27	CTR1, ERCC1 and TS ↑; G2/M cell cycle arrest	[92]
EGFR inhibitor	Gefitinib	Continuous	Head and neck cancer	SCC-1	p-EGFR, MAPK, AKT and STAT3 ↑	[86]
			Lung cancer	A549	N/A	[88]
				A549 and PC9	miR-342-3p ↑	[142]
				A549 and PC9	Circ_MACF1 ↓	[143]
		Episodic	Lung cancer	PC9	HER3 and AKT ↓, HER2 dimerization ↓, EGFR/HER2 and EGFR/HER3 heterodimer formation ↑, ratio of EGFR heterodimer to homodimer ↑	[85]
				H1975	Vimentin ↑, E-cadherin ↓, G0/G1 cell cycle arrest	[87]
	Erlotinib	Continuous	Head and neck	SCC-1	p-EGFR, MAPK, AKT and STAT3 ↑	[86]
			Lung cancer	HCC827	E-cadherin ↓, laminA/C proteins ↓, N- cadherin ↑, vimentin ↑, SLUG ↑, ZEB1 ↑	[116]
				HCC827	PHGDH ↑, ECAR (extracellular acidification rate) ↑	[117]
	Cetuximab	Continuous	Head and neck	LICR-HN2, LICR-HN5, and SC263	Vimentin↑, fibronectin ↑, *ABCG1* ↓, *TP63* ↓, *ALDH1A1* ↓, *ALDH3A1* ↓, *ABCA1* ↓, *SOX21* ↓	[118]
				SCC-1	p-EGFR, MAPK, AKT and STAT3 ↑	[86]
Multiple kinase inhibitor	Lenvatinib	Continuous	Hepatocellular carcinoma	HuH7 and PLC/PRF/5	P-gp ↑, EGF ↑, RTK family proteins ↑	[144]
Proteasome inhibitor	Bortezomib	Continuous	Multiple myeloma	U266	CD56 ↓, CD66a ↓	[113]
				U266	pERK ↑, p-p65 ↑, CD138(-) ↑	[114]
		Episodic		KMS-12-BM	Bcl-2 ↓, Mcl-1 ↓	[115]

* ↓ represent a decrease in the indicated gene, protein, or biological parameter; ↑ represent an increase in the indicated gene, protein, or biological parameter.

## Data Availability

No new data were created or analyzed in this study.

## References

[1] Bray F., Laversanne M., Sung H., Ferlay J., Siegel R.L., Soerjomataram I., Jemal A. (2024). Global cancer statistics 2022: GLOBOCAN estimates of incidence and mortality worldwide for 36 cancers in 185 countries. CA Cancer J. Clin..

[2] Bukowski K., Kciuk M., Kontek R. (2020). Mechanisms of multidrug resistance in cancer chemotherapy. Int. J. Mol. Sci..

[3] Abdallah H.M., Al-Abd A.M., El-Dine R.S., El-Halawany A.M. (2015). P-glycoprotein inhibitors of natural origin as potential tumor chemo-sensitizers: A review. J. Adv. Res..

[4] Marques A.V.L., Ruginsk B.E., Prado L.O., de Lima D.E., Daniel I.W., Moure V.R., Valdameri G. (2025). The association of ABC proteins with multidrug resistance in cancer. Biochim. Biophys. Acta (BBA)-Mol. Cell Res..

[5] Grigoreva T., Sagaidak A., Novikova D., Tribulovich V. (2024). New Insights into Chemoresistance Mediated by Mdm2 Inhibitors: The Benefits of Targeted Therapy over Common Cytostatics. Biomedicines.

[6] Staud F., Pavek P. (2005). Breast cancer resistance protein (BCRP/ABCG2). Int. J. Biochem. Cell Biol..

[7] Grigoreva T., Sagaidak A., Novikova D., Tribulovich V. (2022). Implication of ABC transporters in non-proliferative diseases. Eur. J. Pharmacol..

[8] Emran T.B., Shahriar A., Mahmud A.R., Rahman T., Abir M.H., Siddiquee M.F., Ahmed H., Rahman N., Nainu F., Wahyudin E. (2022). Multidrug Resistance in Cancer: Understanding Molecular Mechanisms, Immunoprevention and Therapeutic Approaches. Front. Oncol..

[9] Habanjar O., Diab-Assaf M., Caldefie-Chezet F., Delort L. (2021). 3D Cell Culture Systems: Tumor Application, Advantages, and Disadvantages. Int. J. Mol. Sci..

[10] Dolznig H., Walzl A., Kramer N., Rosner M., Garin-Chesa P., Hengstschläger M. (2011). Organotypic spheroid cultures to study tumor–stroma interaction during cancer development. Drug Discov. Today Dis. Models.

[11] Hachey S.J., Movsesyan S., Nguyen Q.H., Burtin-Sojo G., Tankazyan A., Wu J., Hoang T., Zhao D., Wang S., Hatch M.M. (2021). An in vitro vascularized micro-tumor model of human colorectal cancer recapitulates in vivo responses to standard-of-care therapy. Lab Chip.

[12] Sobrino A., Phan D.T., Datta R., Wang X., Hachey S.J., Romero-López M., Gratton E., Lee A.P., George S.C., Hughes C.C. (2016). 3D microtumors in vitro supported by perfused vascular networks. Sci. Rep..

[13] Shirure V.S., Hughes C.C.W., George S.C. (2021). Engineering Vascularized Organoid-on-a-Chip Models. Annu. Rev. Biomed. Eng..

[14] Ryan S.L., Baird A.M., Vaz G., Urquhart A.J., Senge M., Richard D.J., O’Byrne K.J., Davies A.M. (2016). Drug Discovery Approaches Utilizing Three-Dimensional Cell Culture. Assay Drug Dev. Technol..

[15] Grigoreva T.A., Kindt D.N., Sagaidak A.V., Novikova D.S., Tribulovich V.G. (2025). Cellular Systems for Colorectal Stem Cancer Cell Research. Cells.

[16] Kadomoto S., Shelley G., Mizokami A., Keller E.T. (2025). Development of Drug-resistant Cell Lines for Experimental Procedures. J. Vis. Exp..

[17] Takeda M., Mizokami A., Mamiya K., Li Y.Q., Zhang J., Keller E.T., Namiki M. (2007). The establishment of two paclitaxel-resistant prostate cancer cell lines and the mechanisms of paclitaxel resistance with two cell lines. Prostate.

[18] Sobue S., Mizutani N., Aoyama Y., Kawamoto Y., Suzuki M., Nozawa Y., Ichihara M., Murate T. (2016). Mechanism of paclitaxel resistance in a human prostate cancer cell line, PC3-PR, and its sensitization by cabazitaxel. Biochem. Biophys. Res. Commun..

[19] Kato T., Fujita Y., Nakane K., Mizutani K., Terazawa R., Ehara H., Kanimoto Y., Kojima T., Nozawa Y., Deguchi T. (2013). CCR1/CCL5 interaction promotes invasion of taxane-resistant PC3 prostate cancer cells by increasing secretion of MMPs 2/9 and by activating ERK and Rac signaling. Cytokine.

[20] Zhang W., Cai J., Chen S., Zheng X., Hu S., Dong W., Lu J., Xing J., Dong Y. (2015). Paclitaxel resistance in MCF-7/PTX cells is reversed by paeonol through suppression of the SET/phosphatidylinositol 3-kinase/Akt pathway. Mol. Med. Rep..

[21] Aniogo E.C., George B.P., Abrahamse H. (2022). Characterization of resistant MCF-7 breast cancer cells developed by repeated cycles of photodynamic therapy. Front. Pharmacol..

[22] Karimifard S.A., Salehzadeh-Yazdi A., Taghizadeh-Tabarsi R., Akbari-Birgani S. (2024). Mechanical effects modulate drug resistance in MCF-7-derived organoids: Insights into the wnt/β-catenin pathway. Biochem. Biophys. Res. Commun..

[23] Pan X., Hong X., Li S., Meng P., Xiao F. (2021). METTL3 promotes adriamycin resistance in MCF-7 breast cancer cells by accelerating pri-microRNA-221-3p maturation in a m6A-dependent manner. Exp. Mol. Med..

[24] Wang H., Vo T., Hajar A., Li S., Chen X., Parissenti A.M., Brindley D.N., Wang Z. (2014). Multiple mechanisms underlying acquired resistance to taxanes in selected docetaxel-resistant MCF-7 breast cancer cells. BMC Cancer.

[25] Angelucci A., Mari M., Millimaggi D., Giusti I., Carta G., Bologna M., Dolo V. (2010). Suberoylanilide hydroxamic acid partly reverses resistance to paclitaxel in human ovarian cancer cell lines. Gynecol. Oncol..

[26] Kim Y., Yun H.J., Choi K.H., Kim C.W., Lee J.H., Weicker R., Kim S.M., Park K.C. (2023). Discovery of New Anti-Cancer Agents against Patient-Derived Sorafenib-Resistant Papillary Thyroid Cancer. Int. J. Mol. Sci..

[27] Sequeira D., Baptista P.V., Valente R., Piedade M.F.M., Garcia M.H., Morais T.S., Fernandes A.R. (2021). Cu(I) complexes as new antiproliferative agents against sensitive and doxorubicin resistant colorectal cancer cells: Synthesis, characterization, and mechanisms of action. Dalton Trans..

[28] Maleki Vareki S., Salim K.Y., Danter W.R., Koropatnick J. (2018). Novel anti-cancer drug COTI-2 synergizes with therapeutic agents and does not induce resistance or exhibit cross-resistance in human cancer cell lines. PLoS ONE.

[29] Greaves M., Maley C.C. (2012). Clonal evolution in cancer. Nature.

[30] Mohammad R.M., Muqbil I., Lowe L., Yedjou C., Hsu H.Y., Lin L.T., Siegelin M.D., Fimognari C., Kumar N.B., Dou Q.P. (2015). Broad targeting of resistance to apoptosis in cancer. Semin. Cancer Biol..

[31] Babaei G., Aziz S.G., Jaghi N.Z.Z. (2021). EMT, cancer stem cells and autophagy; The three main axes of metastasis. Biomed. Pharmacother..

[32] Rubtsova S.N., Zhitnyak I.Y., Gloushankova N.A. (2022). Dual role of E-cadherin in cancer cells. Tissue Barriers.

[33] Uba A.I., Bui-Linh C., Thornton J.M., Olivieri M., Wu C. (2023). Computational analysis of drug resistance of taxanes bound to human β-tubulin mutant (D26E). J. Mol. Graph. Model..

[34] Holohan C., Van Schaeybroeck S., Longley D.B., Johnston P.G. (2013). Cancer drug resistance: An evolving paradigm. Nat. Rev. Cancer.

[35] Ikram M., Uddin Z., Shehzad A. (2022). Biomarkers in Cancer Survival and Drug Resistance. Cancer Biomarkers in Diagnosis and Therapeutics.

[36] Barathan M., Zulpa A.K., Ng S.L., Lokanathan Y., Ng M.H., Law J.X. (2024). Innovative Strategies to Combat 5-Fluorouracil Resistance in Colorectal Cancer: The Role of Phytochemicals and Extracellular Vesicles. Int. J. Mol. Sci..

[37] Sethy C., Kundu C.N. (2021). 5-Fluorouracil (5-FU) resistance and the new strategy to enhance the sensitivity against cancer: Implication of DNA repair inhibition. Biomed. Pharmacother..

[38] Liao J., Qin Q.H., Lv F.Y., Huang Z., Lian B., Wei C.Y., Mo Q.G., Tan Q.X. (2023). IKKα inhibition re-sensitizes acquired adriamycin-resistant triple negative breast cancer cells to chemotherapy-induced apoptosis. Sci. Rep..

[39] Zhang X., Yashiro M., Qiu H., Nishii T., Matsuzaki T., Hirakawa K. (2010). Establishment and characterization of multidrug-resistant gastric cancer cell lines. Anticancer Res..

[40] Yang R., Guo Z., Zhao Y., Ma L., Li B., Yang C. (2021). Compound 968 reverses adriamycin resistance in breast cancer MCF-7ADR cells via inhibiting P-glycoprotein function independently of glutaminase. Cell Death Discov..

[41] Oh Y., Lee J.S., Lee J.S., Park J.H., Kim H.S., Yoon S. (2022). JAK2 Inhibitor, Fedratinib, Inhibits P-gp Activity and Co-Treatment Induces Cytotoxicity in Antimitotic Drug-Treated P-gp Overexpressing Resistant KBV20C Cancer Cells. Int. J. Mol. Sci..

[42] Grigoreva T.A., Sagaidak A.V., Vorona S.V., Novikova D.S., Tribulovich V.G. (2022). ATP Mimetic Attack on the Nucleotide-Binding Domain to Overcome ABC Transporter Mediated Chemoresistance. ACS Med. Chem. Lett..

[43] Grigoreva T., Romanova A., Sagaidak A., Vorona S., Novikova D., Tribulovich V. (2020). Mdm2 inhibitors as a platform for the design of P-glycoprotein inhibitors. Bioorg. Med. Chem. Lett..

[44] Lu Y., Bian D., Zhang X., Zhang H., Zhu Z. (2021). Inhibition of Bcl-2 and Bcl-xL overcomes the resistance to the third-generation EGFR tyrosine kinase inhibitor osimertinib in non-small cell lung cancer. Mol. Med. Rep..

[45] Lamichhane A., Tavana H. (2024). Three-Dimensional Tumor Models to Study Cancer Stemness-Mediated Drug Resistance. Cell. Mol. Bioeng..

[46] Nikdouz A., Orso F. (2023). Emerging roles of 3D-culture systems in tackling tumor drug resistance. Cancer Drug Resist..

[47] Becker J.L., Blanchard D.K. (2007). Characterization of primary breast carcinomas grown in three-dimensional cultures. J. Surg. Res..

[48] Breslin S., O’Driscoll L. (2013). Three-dimensional cell culture: The missing link in drug discovery. Drug Discov. Today..

[49] Amaral R.L.F., Miranda M., Marcato P.D., Swiech K. (2017). Comparative analysis of 3D bladder tumor spheroids obtained by forced floating and hanging drop methods for drug screening. Front. Physiol..

[50] Petersen O.W., Rønnov-Jessen L., Howlett A.R., Bissell M.J. (1992). Interaction with Basement Membrane Serves to Rapidly Distinguish Growth and Differentiation Pattern of Normal and Malignant Human Breast Epithelial Cells. Proc. Natl. Acad. Sci. USA.

[51] Ramzy G.M., Koessler T., Ducrey E., McKee T., Ris F., Buchs N., Rubbia-Brandt L., Dietrich P.Y., Nowak-Sliwinska P. (2020). Patient-derived in vitro models for drug discovery in colorectal carcinoma. Cancers.

[52] Dontu G., Abdallah W.M., Foley J.M., Jackson K.W., Clarke M.F., Kawamura M.J., Wicha M.S. (2003). In vitro propagation and transcriptional profiling of human mammary stem/progenitor cells. Genes Dev..

[53] Ponti D., Costa A., Zaffaroni N., Pratesi G., Petrangolini G., Coradini D., Pilotti S., Pierotti M.A., Diadone M.G. (2005). Isolation and in vitro propagation of tumorigenic breast cancer cells with stem/progenitor cell properties. Cancer Res..

[54] Costa E.C., Moreira A.F., de Melo-Diogo D., Gaspar V.M., Carvalho M.P., Correia I.J. (2016). 3D tumor spheroids: An overview on the tools and techniques used for their analysis. Biotechnol. Adv..

[55] Hutchinson L., Kirk R. (2011). High drug attrition rates-where are we going wrong?. Nat. Rev. Clin. Oncol..

[56] Bahmad H.F., Cheaito K., Chalhoub R.M., Hadadeh O., Monzer A., Ballout F., El-Hajj A., Mukherji D., Liu Y.N., Daoud G. (2018). Sphere-Formation Assay: Three-Dimensional in vitro Culturing of Prostate Cancer Stem/Progenitor Sphere-Forming Cells. Front. Oncol..

[57] Costa E.C., de Melo-Diogo D., Moreira A.F., Carvalho M.P., Correia I.J. (2018). Spheroids formation on non-adhesive surfaces by liquid overlay technique: Considerations and practical approaches. Biotechnol. J..

[58] Achilli T.M., Meyer J., Morgan J.R. (2012). Advances in the formation, use and understanding of multi-cellular spheroids. Expert. Opin. Biol. Ther..

[59] Carpenedo R.L., Sargent C.Y., McDevitt T.C. (2007). Rotary suspension culture enhances the efficiency, yield, and homogeneity of embryoid body differentiation. Stem Cells.

[60] Smart C.E., Morrison B.J., Saunus J.M., Vargas A.C., Keith P., Reid L., Wockner L., Amiri M.A., Sarkar D., Simpson P.T. (2013). In Vitro analysis of breast cancer cell line tumourspheres and primary human breast epithelia mammospheres demonstrates inter- and intrasphere heterogeneity. PLoS ONE.

[61] Yu M., Bardia A., Aceto N., Bersani F., Madden M.W., Donaldson M.C., Deasi R., Zhu H., Comaills V., Zheng Z. (2014). Cancer Therapy. Ex Vivo culture of circulating breast tumor cells for individualized testing of drug susceptibility. Science.

[62] Mun S.J., Ryu J.S., Lee M.O., Son Y.S., Oh S.J., Cho H.S., Son M.Y., Kim D.S., Kim S.J., Yoo H.J. (2019). Generation of expandable human pluripotent stem cell-derived hepatocyte-like liver organoids. J. Hepatol..

[63] Drost J., Karthaus W.R., Gao D., Driehuis E., Sawyers C.L., Chen Y., Clevers H. (2016). Organoid culture systems for prostate epithelial and cancer tissue. Nat. Protoc..

[64] Broutier L., Andersson-Rolf A., Hindley C.J., Boj S.F., Clevers H., Koo B.K., Huch M. (2016). Culture and establishment of self-renewing human and mouse adult liver and pancreas 3D organoids and their genetic manipulation. Nat. Protoc..

[65] Driehuis E., Clevers H. (2017). CRISPR/Cas 9 genome editing and its applications in organoids. Am. J. Physiol.-Gastrointest. Liver Physiol..

[66] Langhans S.A. (2018). Three-Dimensional in Vitro Cell Culture Models in Drug Discovery and Drug Repositioning. Front. Pharmacol..

[67] Fiore D., Di Giacomo F., Kyriakides P., Inghirami G. (2017). Patient-Derived-Tumor-Xenograft: Modeling cancer for basic and translational cancer research. Clin. Diagn. Pathol..

[68] Kaur R., Bhardwaj A., Gupta S. (2023). Cancer treatment therapies: Traditional to modern approaches to combat cancers. Mol. Biol. Rep..

[69] Wang J.J., Lei K.F., Han F. (2018). Tumor microenvironment: Recent advances in various cancer treatments. Eur. Rev. Med. Pharmacol. Sci..

[70] Aliseda D., Arredondo J., Sánchez-Justicia C., Alvarellos A., Rodríguez J., Matos I., Rotellar F., Baixauli J., Pastor C. (2024). Survival and safety after neoadjuvant chemotherapy or upfront surgery for locally advanced colon cancer: Meta-analysis. Br. J. Surg..

[71] Godfroy M., Loaec C., Berton D., Guérin-Charbonnel C., Classe J.M. (2023). Impact of consolidation chemotherapy after delayed complete surgery in advanced epithelial ovarian cancer: A propensity score analysis. Int. J. Gynecol. Cancer.

[72] Hank T., Klaiber U., Hinz U., Schütte D., Leonhardt C.S., Bergmann F., Hackert T., Jäger D., Büchler M.W., Strobel O. (2023). Oncological Outcome of Conversion Surgery After Preoperative Chemotherapy for Metastatic Pancreatic Cancer. Ann. Surg..

[73] Tilsed C.M., Fisher S.A., Nowak A.K., Lake R.A., Lesterhuis W.J. (2022). Cancer chemotherapy: Insights into cellular and tumor microenvironmental mechanisms of action. Front. Oncol..

[74] Schmidt E.V., Chisamore M.J., Chaney M.F., Maradeo M.E., Anderson J., Baltus G.A., Pinheiro E.M., Uebele V.N. (2020). Assessment of clinical activity of PD-1 checkpoint inhibitor combination therapies reported in clinical trials. JAMA Netw. Open.

[75] van Hagen P., Hulshof M.C., van Lanschot J.J., Steyerberg E.W., van Berge Henegouwen M.I., Wijnhoven B.P., Richel D.J., Nieuwenhuijzen G.A., Hospers G.A., Bonenkamp J.J. (2012). Preoperative chemoradiotherapy for esophageal or junctional cancer. N. Engl. J. Med..

[76] Achilli P., Crippa J., Grass F., Mathis K.L., D’Angelo A.D., Abd El Aziz M.A., Day C.N., Harmsen W.S., Larson D.W. (2021). Survival impact of adjuvant chemotherapy in patients with stage IIA colon cancer: Analysis of the National Cancer Database. Int. J. Cancer.

[77] Basourakos S.P., Li L., Aparicio A.M., Corn P.G., Kim J., Thompson T.C. (2017). Combination platinum-based and DNA damage response-targeting cancer therapy: Evolution and future directions. Curr. Med. Chem..

[78] Schiff P.B., Fant J., Horwitz S.B. (1979). Promotion of microtubule assembly in vitro by taxol. Nature.

[79] Tewey K.M., Rowe T.C., Yang L., Halligan B.D., Liu L.F. (1984). Adriamycin-induced DNA damage mediated by mammalian DNA topoisomerase II. Science.

[80] DeVita V.T., Lawrence T.S., Rosenberg S.A. (2008). Devita, Hellman, and Rosenberg’s Cancer: Principles & Practice of Oncology.

[81] Lee Y.T., Tan Y.J., Oon C.E. (2018). Molecular targeted therapy: Treating cancer with specificity. Eur. J. Pharmacol..

[82] Min H.Y., Lee H.Y. (2022). Molecular targeted therapy for anticancer treatment. Exp. Mol. Med..

[83] Bayat Mokhtari R., Homayouni T.S., Baluch N., Morgatskaya E., Kumar S., Das B., Yeger H. (2017). Combination therapy in combating cancer. Oncotarget.

[84] Kolbeinsson H.M., Chandana S., Wright G.P., Chung M. (2023). Pancreatic Cancer: A Review of Current Treatment and Novel Therapies. J. Investig. Surg..

[85] Koizumi F., Shimoyama T., Taguchi F., Saijo N., Nishio K. (2005). Establishment of a human non-small cell lung cancer cell line resistant to gefitinib. Int. J. Cancer.

[86] Benavente S., Huang S., Armstrong E.A., Chi A., Hsu K.T., Wheeler D.L., Harari P.M. (2009). Establishment and characterization of a model of acquired resistance to epidermal growth factor receptor targeting agents in human cancer cells. Clin. Cancer Res..

[87] Zhao B.X., Wang J., Song B., Wei H., Lv W.P., Tian L.M., Li M., Lv S. (2015). Establishment and biological characteristics of acquired gefitinib resistance in cell line NCI-H1975/gefinitib-resistant with epidermal growth factor receptor T790M mutation. Mol. Med. Rep..

[88] Panda M., Tripathi S.K., Biswal B.K. (2020). Plumbagin promotes mitochondrial mediated apoptosis in gefitinib sensitive and resistant A549 lung cancer cell line through enhancing reactive oxygen species generation. Mol. Biol. Rep..

[89] Grigoreva T., Sagaidak A., Romanova A., Novikova D., Garabadzhiu A., Tribulovich V. (2021). Establishment of drug-resistant cell lines under the treatment with chemicals acting through different mechanisms. Chem. Biol. Interact..

[90] Amaral M.V.S., DE Sousa Portilho A.J., DA Silva E.L., DE Oliveira Sales L., DA Silva Maués J.H., DE Moraes M.E.A., Moreira-Nunes C.A. (2019). Establishment of Drug-resistant Cell Lines as a Model in Experimental Oncology: A Review. Anticancer Res..

[91] Han T., Zhu X., Wang J., Zhao H., Ma Q., Zhao J., Qiu X., Fan Q. (2014). Establishment and characterization of a cisplatin-resistant human osteosarcoma cell line. Oncol. Rep..

[92] Govindan S.V., Kulsum S., Pandian R.S., Das D., Seshadri M., Hicks W., Kuriakose M.A., Suresh A. (2015). Establishment and characterization of triple drug resistant head and neck squamous cell carcinoma cell lines. Mol. Med. Rep..

[93] Zhou Y., Ling X.L., Li S.W., Li X.Q., Yan B. (2010). Establishment of a human hepatoma multidrug resistant cell line in vitro. World J. Gastroenterol..

[94] Nitta A., Chung Y.S., Nakata B., Yashiro M., Onoda N., Maeda K., Sawada T., Sowa M. (1997). Establishment of a cisplatin-resistant gastric carcinoma cell line OCUM-2M/DDP. Cancer Chemother. Pharmacol..

[95] Noordhuis P., Laan A.C., van de Born K., Honeywell R.J., Peters G.J. (2019). Coexisting Molecular Determinants of Acquired Oxaliplatin Resistance in Human Colorectal and Ovarian Cancer Cell Lines. Int. J. Mol. Sci..

[96] Shi C.J., Xue Z.H., Zeng W.Q., Deng L.Q., Pang F.X., Zhang F.W., Fu W.M., Zhang J.F. (2023). LncRNA-NEF suppressed oxaliplatin resistance and epithelial-mesenchymal transition in colorectal cancer through epigenetically inactivating MEK/ERK signaling. Cancer Gene Ther..

[97] Zeng K., Li W., Wang Y., Zhang Z., Zhang L., Zhang W., Xing Y., Zhou C. (2023). Inhibition of CDK1 Overcomes Oxaliplatin Resistance by Regulating ACSL4-mediated Ferroptosis in Colorectal Cancer. Adv. Sci..

[98] Chen S.Y., Hu S.S., Dong Q., Cai J.X., Zhang W.P., Sun J.Y., Wang T.T., Xie J., He H.R., Xing J.F. (2013). Establishment of paclitaxel-resistant breast cancer cell line and nude mice models, and underlying multidrug resistance mechanisms in vitro and in vivo. Asian Pac. J. Cancer Prev..

[99] Němcová-Fürstová V., Kopperová D., Balušíková K., Ehrlichová M., Brynychová V., Václavíková R., Daniel P., Souček P., Kovář J. (2016). Characterization of acquired paclitaxel resistance of breast cancer cells and involvement of ABC transporters. Toxicol. Appl. Pharmacol..

[100] Niu B.H., Wang J.J., Xi Y., Ji X.Y. (2010). The establishment and characterization of adriamycin-resistant cell lines derived from Saos-2. Med. Sci. Monit..

[101] Yu D.S., Ma C.P., Chang S.Y. (2000). Establishment and characterization of renal cell carcinoma cell lines with multidrug resistance. Urol. Res..

[102] Uchiyama-Kokubu N., Watanabe T. (2001). Establishment and characterization of adriamycin-resistant human colorectal adenocarcinoma HCT-15 cell lines with multidrug resistance. Anticancer Drugs.

[103] Kiura K., Ohnoshi T., Tabata M., Shibayama T., Kimura I. (1993). Establishment of an adriamycin-resistant subline of human small cell lung cancer showing multifactorial mechanisms of resistance. Acta Med. Okayama.

[104] Greife A., Tukova J., Steinhoff C., Scott S.D., Schulz W.A., Hatina J. (2015). Establishment and characterization of a bladder cancer cell line with enhanced doxorubicin resistance by mevalonate pathway activation. Tumour Biol..

[105] Guo B., Villeneuve D.J., Hembruff S.L., Kirwan A.F., Blais D.E., Bonin M., Parissenti A.M. (2004). Cross-resistance studies of isogenic drug-resistant breast tumor cell lines support recent clinical evidence suggesting that sensitivity to paclitaxel may be strongly compromised by prior doxorubicin exposure. Breast Cancer Res. Treat..

[106] Kimiya K., Naito S., Soejima T., Sakamoto N., Kotoh S., Kumazawa J., Tsuruo T. (1992). Establishment and characterization of doxorubicin-resistant human bladder cancer cell line, KK47/ADM. J. Urol..

[107] Oda Y., Matsumoto Y., Harimaya K., Iwamoto Y., Tsuneyoshi M. (2000). Establishment of new multidrug-resistant human osteosarcoma cell lines. Oncol. Rep..

[108] Harada K., Ferdous T., Ueyama Y. (2014). Establishment of 5-fluorouracil-resistant oral squamous cell carcinoma cell lines with epithelial to mesenchymal transition changes. Int. J. Oncol..

[109] Takahashi K., Tanaka M., Inagaki A., Wanibuchi H., Izumi Y., Miura K., Nagayama K., Shiota M., Iwao H. (2013). Establishment of a 5-fluorouracil-resistant triple-negative breast cancer cell line. Int. J. Oncol..

[110] Wattanawongdon W., Hahnvajanawong C., Namwat N., Kanchanawat S., Boonmars T., Jearanaikoon P., Leelayuwat C., Techasen A., Seubwai W. (2015). Establishment and characterization of gemcitabine-resistant human cholangiocarcinoma cell lines with multidrug resistance and enhanced invasiveness. Int. J. Oncol..

[111] Chiu C.H., Lin Y.J., Ramesh S., Kuo W.W., Chen M.C., Kuo C.H., Li C.C., Wang T.F., Lin Y.M., Liao P.H. (2023). Gemcitabine resistance in non-small cell lung cancer is mediated through activation of the PI3K/AKT/NF-κB pathway and suppression of ERK signaling by reactive oxygen species. J. Biochem. Mol. Toxicol..

[112] Wang C., Zhang W., Fu M., Yang A., Huang H., Xie J. (2015). Establishment of human pancreatic cancer gemcitabine-resistant cell line with ribonucleotide reductase overexpression. Oncol. Rep..

[113] Baughn L.B., Sachs Z., Noble-Orcutt K.E., Mitra A., Van Ness B.G., Linden M.A. (2017). Phenotypic and functional characterization of a bortezomib-resistant multiple myeloma cell line by flow and mass cytometry. Leuk. Lymphoma.

[114] Park J., Bae E.K., Lee C., Choi J.H., Jung W.J., Ahn K.S., Yoon S.S. (2014). Establishment and characterization of bortezomib-resistant U266 cell line: Constitutive activation of NF-κB-mediated cell signals and/or alterations of ubiquitylation-related genes reduce bortezomib-induced apoptosis. BMB Rep..

[115] Downey-Kopyscinski S.L., Srinivasa S., Kisselev A.F. (2022). A clinically relevant pulse treatment generates a bortezomib-resistant myeloma cell line that lacks proteasome mutations and is sensitive to Bcl-2 inhibitor venetoclax. Sci. Rep..

[116] Hu C., Zhou A., Hu X., Xiang Y., Huang M., Huang J., Yang D., Tang Y. (2022). LMNA reduced acquired resistance to erlotinib in NSCLC by reversing the epithelial-mesenchymal transition via the FGFR/MAPK/c-fos signaling pathway. Int. J. Mol. Sci..

[117] Dong J.K., Lei H.M., Liang Q., Tang Y.B., Zhou Y., Wang Y., Zhang S., Li W.B., Tong Y., Zhuang G. (2018). Overcoming erlotinib resistance in EGFR mutation-positive lung adenocarcinomas through repression of phosphoglycerate dehydrogenase. Theranostics.

[118] Boeckx C., Blockx L., de Beeck K.O., Limame R., Camp G.V., Peeters M., Vermorken J.B., Specenier P., Wouters A., Baay M. (2015). Establishment and characterization of cetuximab resistant head and neck squamous cell carcinoma cell lines: Focus on the contribution of the AP-1 transcription factor. Am. J. Cancer Res..

[119] Tsou S.H., Chen T.M., Hsiao H.T., Chen Y.H. (2015). A critical dose of doxorubicin is required to alter the gene expression profiles in MCF-7 cells acquiring multidrug resistance. PLoS ONE.

[120] Kot M., Simiczyjew A., Wądzyńska J., Ziętek M., Matkowski R., Nowak D. (2024). Characterization of two melanoma cell lines resistant to BRAF/MEK inhibitors (vemurafenib and cobimetinib). Cell Commun. Signal..

[121] Sciarrillo R., Wojtuszkiewicz A., Kooi I.E., Gómez V.E., Boggi U., Jansen G., Kaspers G.J., Cloos J., Giovannetti E. (2016). Using RNA-sequencing to Detect Novel Splice Variants Related to Drug Resistance in In Vitro Cancer Models. J. Vis. Exp..

[122] Han E.K., Tahir S.K., Cherian S.P., Collins N., Ng S.C. (2000). Modulation of paclitaxel resistance by annexin IV in human cancer cell lines. Br. J. Cancer.

[123] Wen J., Zheng B., Hu Y., Zhang X., Yang H., Luo K.J., Zhang X., Li Y.F., Fu J.H. (2009). Establishment and biological analysis of the EC109/CDDP multidrug-resistant esophageal squamous cell carcinoma cell line. Oncol. Rep..

[124] Samli H., Samli M., Vatansever B., Ardicli S., Aztopal N., Dincel D., Sahin A., Balci F. (2019). Paclitaxel resistance and the role of miRNAs in prostate cancer cell lines. World J. Urol..

[125] Wen J., Yeo S., Wang C., Chen S., Sun S., Haas M.A., Tu W., Jin F., Guan J.L. (2015). Autophagy inhibition re-sensitizes pulse stimulation-selected paclitaxel-resistant triple negative breast cancer cells to chemotherapy-induced apoptosis. Breast Cancer Res. Treat..

[126] Krzywik J., Aminpour M., Maj E., Mozga W., Wietrzyk J., Tuszyński J.A., Huczyński A. (2020). New Series of Double-Modified Colchicine Derivatives: Synthesis, Cytotoxic Effect and Molecular Docking. Molecules.

[127] Lahmar A., Mathey A., Aires V., Elgueder D., Vejux A., Khlifi R., Sioud F., Chekir-Ghedira L., Delmas D. (2021). Essential Oils, *Pituranthos chloranthus* and *Teucrium ramosissimum*, Chemosensitize Resistant Human Uterine Sarcoma MES-SA/Dx5 Cells to Doxorubicin by Inducing Apoptosis and Targeting P-Glycoprotein. Nutrients.

[128] Coley H.M., Labeed F.H., Thomas H., Hughes M.P. (2007). Biophysical characterization of MDR breast cancer cell lines reveals the cytoplasm is critical in determining drug sensitivity. Biochim. Biophys. Acta (BBA)-Gen. Subj..

[129] McDermott M., Eustace A.J., Busschots S., Breen L., Crown J., Clynes M., O’Donovan N., Stordal B. (2014). In vitro Devel-opment of Chemotherapy and Targeted Therapy Drug-Resistant Cancer Cell Lines: A Practical Guide with Case Studies. Front. Oncol..

[130] Packeiser E.M., Engels L., Nolte I., Goericke-Pesch S., Murua Escobar H. (2023). MDR1 Inhibition Reverses Doxorubi-cin-Resistance in Six Doxorubicin-Resistant Canine Prostate and Bladder Cancer Cell Lines. Int. J. Mol. Sci..

[131] Morshneva A.V., Gnedina O.O., Kindt D.N., Igotti M.V. (2022). Establishment and Characterization of Human Colon-Cancer Cells Resistant to Cisplatin. Cell Tissue Biol..

[132] Iwasaki I., Sugiyama H., Kanazawa S., Hemmi H. (2002). Establishment of cisplatin-resistant variants of human neuroblastoma cell lines, TGW and GOTO, and their drug cross-resistance profiles. Cancer Chemother. Pharmacol..

[133] Shibata K., Umezu T., Sakurai M., Kajiyama H., Yamamoto E., Ino K., Nawa A., Kikkawa F. (2011). Establishment of cis-platin-resistant ovarian yolk sac tumor cells and investigation of the mechanism of cisplatin resistance using this cell line. Gynecol. Obstet. Investig..

[134] Tang H., Liu Y.J., Liu M., Li X. (2007). Establishment and gene analysis of an oxaliplatin-resistant colon cancer cell line THC8307/L-OHP. Anticancer Drugs.

[135] Zuo K.Q., Zhang X.P., Zou J., Li D., Lv Z.W. (2010). Establishment of a paclitaxel resistant human breast cancer cell strain (MCF-7/Taxol) and intracellular paclitaxel binding protein analysis. J. Int. Med. Res..

[136] Liu Z.H., He Y.P., Zhou Y., Zhang P., Qin H. (2011). Establishment and identification of the human multi-drug-resistant cholangiocarcinoma cell line QBC939/ADM. Mol. Biol. Rep..

[137] Środa-Pomianek K., Michalak K., Palko-Łabuz A., Uryga A., Świątek P., Majkowski M., Wesołowska O. (2019). The Combined Use of Phenothiazines and Statins Strongly Affects Doxorubicin-Resistance, Apoptosis, and Cox-2 Activity in Colon Cancer Cells. Int. J. Mol. Sci..

[138] Mallappa S., Neeli P.K., Karnewar S., Kotamraju S. (2019). Doxorubicin induces prostate cancer drug resistance by upregulation of ABCG4 through GSH depletion and CREB activation: Relevance of statins in chemosensitization. Mol. Carcinog..

[139] Uchibori K., Kasamatsu A., Sunaga M., Yokota S., Sakurada T., Kobayashi E., Yoshikawa M., Uzawa K., Ueda S., Tanzawa H. (2012). Establishment and characterization of two 5-fluorouracil-resistant hepatocellular carcinoma cell lines. Int. J. Oncol..

[140] Kim M.J., Kim H.S., Kang H.W., Lee D.E., Hong W.C., Kim J.H., Kim M., Cheong J.H., Kim H.J., Park J.S. (2023). SLC38A5 Modulates Ferroptosis to Overcome Gemcitabine Resistance in Pancreatic Cancer. Cells.

[141] She C., Wu C., Guo W., Xie Y., Li S., Liu W., Xu C., Li H., Cao P., Yang Y. (2023). Combination of RUNX1 inhibitor and gemcitabine mitigates chemo-resistance in pancreatic ductal adenocarcinoma by modulating BiP/PERK/eIF2α-axis-mediated endoplasmic reticulum stress. J. Exp. Clin. Cancer Res..

[142] Zheng F., Zhang H., Lu J. (2019). Identification of potential microRNAs and their targets in promoting gefitinib resistance by integrative network analysis. J. Thorac. Dis..

[143] Fan D., Yang Y., Zhang W. (2022). A novel circ_MACF1/miR-942-5p/TGFBR2 axis regulates the functional behaviors and drug sensitivity in gefitinib-resistant non-small cell lung cancer cells. BMC Pulm. Med..

[144] Hu B., Zou T., Qin W., Shen X., Su Y., Li J., Chen Y., Zhang Z., Sun H., Zheng Y. (2022). Inhibition of EGFR overcomes acquired lenvatinib resistance driven by STAT3-ABCB1 signaling in hepatocellular carcinoma. Cancer Res..

[145] Mikuła-Pietrasik J., Witucka A., Pakuła M., Uruski P., Begier-Krasińska B., Niklas A., Tykarski A., Książek K. (2019). Comprehensive review on how platinum- and taxane-based chemotherapy of ovarian cancer affects biology of normal cells. Cell. Mol. Life Sci..

[146] Davies K.J., Doroshow J.H. (1986). Redox cycling of anthracyclines by cardiac mitochondria. I. Anthracycline radical formation by NADH dehydrogenase. J. Biol. Chem..

[147] Wang Y., Li C., Jiang T., Yin Y., Wang Y., Zhao H., Yu L. (2024). A comprehensive exploration of twist1 to identify a biomarker for tumor immunity and prognosis in pan-cancer. Medicine.

[148] Mini E., Nobili S., Caciagli B., Landini I., Mazzei T. (2006). Cellular pharmacology of gemcitabine. Ann. Oncol..

[149] Uribe M.L., Marrocco I., Yarden Y. (2021). EGFR in Cancer: Signaling Mechanisms, Drugs, and Acquired Resistance. Cancers.

[150] Ramírez Moreno M., Bulgakova N.A. (2022). The Cross-Talk Between EGFR and E-Cadherin. Front. Cell Dev. Biol..

[151] Allmeroth K., Horn M., Kroef V., Miethe S., Müller R.U., Denzel M.S. (2021). Bortezomib resistance mutations in PSMB5 determine response to second-generation proteasome inhibitors in multiple myeloma. Leukemia.

[152] Kale A.J., Moore B.S. (2012). Molecular mechanisms of acquired proteasome inhibitor resistance. J. Med. Chem..

[153] Ri M., Iida S., Nakashima T., Miyazaki H., Mori F., Ito A., Inagaki A., Kusumoto S., Ishida T., Komatsu H. (2010). Bortezomib-resistant myeloma cell lines: A role for mutated PSMB5 in preventing the accumulation of unfolded proteins and fatal ER stress. Leukemia.

[154] Tsvetkov P., Detappe A., Cai K., Keys H.R., Brune Z., Ying W., Thiru P., Reidy M., Kugener G., Rossen J. (2019). Mitochondrial metabolism promotes adaptation to proteotoxic stress. Nat. Chem. Biol..

[155] Li Y., Zhao B., Peng J., Tang H., Wang S., Peng S., Ye F., Wang J., Ouyang K., Li J. (2024). Inhibition of NF-κB signaling unveils novel strategies to overcome drug resistance in cancers. Drug Resist. Updates.

[156] Januchowski R., Sterzyńska K., Zaorska K., Sosińska P., Klejewski A., Brązert M., Nowicki M., Zabel M. (2016). Analysis of MDR genes expression and cross-resistance in eight drug resistant ovarian cancer cell lines. J. Ovarian Res..

[157] Ukai S., Honma R., Sakamoto N., Yamamoto Y., Pham Q.T., Harada K., Takashima T., Taniyama D., Asai R., Fukada K. (2020). Molecular biological analysis of 5-FU-resistant gastric cancer organoids; KHDRBS3 contributes to the attainment of features of cancer stem cell. Oncogene.

[158] Friedrich J., Seidel C., Ebner R., Kunz-Schughart L.A. (2009). Spheroid-based drug screen: Considerations and practical approach. Nat. Protoc..

